# Necrotizing Pneumonia, a Skeletal Muscle Lesion, and a Fungating Duodenal Mass: An Atypical Presentation of Rapidly Progressing Lung Adenocarcinoma

**DOI:** 10.7759/cureus.53950

**Published:** 2024-02-10

**Authors:** Justin Ferkin, Brooke Williams, Phillip S Moore

**Affiliations:** 1 Surgery, Campbell University School of Osteopathic Medicine, Salisbury, USA; 2 Internal Medicine, Novant Health, Winston-Salem, USA; 3 Vascular Surgery, Novant Health, Winston-Salem, USA

**Keywords:** adenocarcinoma lung, rapid progression, necrotizing pneumonia, skeletal muscle metastasis, non small cell lung cancer, duodenal bulb obstruction, fungating mass, case report

## Abstract

Lung adenocarcinoma, the predominant subtype of non-small cell lung cancer, typically metastasizes to common sites such as the liver and adrenal glands. However, rare instances involve skeletal muscle metastasis. We present a case of a 45-year-old female with a medical history of hypertension, epilepsy, and fibromyalgia, who presented to the emergency department with hemoptysis and multifocal pain. Chest imaging revealed a cavitary lesion which appeared to be necrotizing pneumonia. Further investigations uncovered a fluid collection in the left thigh, which would be identified as poorly differentiated carcinoma. Subsequent testing identified the lung as the primary source of metastasis. Despite radiation treatment, the patient's condition deteriorated over the next 50 days, highlighting the aggressive nature of the disease.

## Introduction

Lung adenocarcinoma is the prevalent subtype of non-small cell lung cancer (NSCLC) in the United States which accounts for 40% of all lung cancer diagnoses [1]. As of 2018, there are approximately 641 new cases of lung cancer diagnosed each day in the United States [2]. The 5-year survival rate of lung cancer was most recently estimated to be 18% [2]. Adenocarcinoma of the lung originates from glandular or secretory cells that line the outer surface of the lungs and produce mucus [1]. Lung adenocarcinoma has the potential to spread both locally and metastatically. Local spread includes the lung pleura, diaphragm, and pericardium with significant disease involving the mediastinum and great vessels [1]. Distant spread often occurs hematogenously to the liver and adrenal glands, with rare occurrence in the skeletal muscle [3]. The following case deviates from the conventional metastatic pattern, involving a rare occurrence of skeletal muscle metastasis. To appreciate the significance of this atypical presentation, it is essential to understand the usual progression of lung adenocarcinoma and the common organs it affects. This case sheds light on the unpredictable nature of the disease and underscores the importance of a comprehensive understanding for both clinicians and researchers.

## Case presentation

**Day 0**: A 45-year-old female with a past medical history of hypertension, epilepsy, and fibromyalgia presented to the emergency department with pain in her left upper extremity, left hip, and abdomen. Physical exam revealed generalized abdominal tenderness, and pain on palpation of left humerus and left anterior thigh. All imaging performed was found to be normal. The patient’s pain was under control and was discharged with a diagnosis of generalized pain.

**Figure 1:**
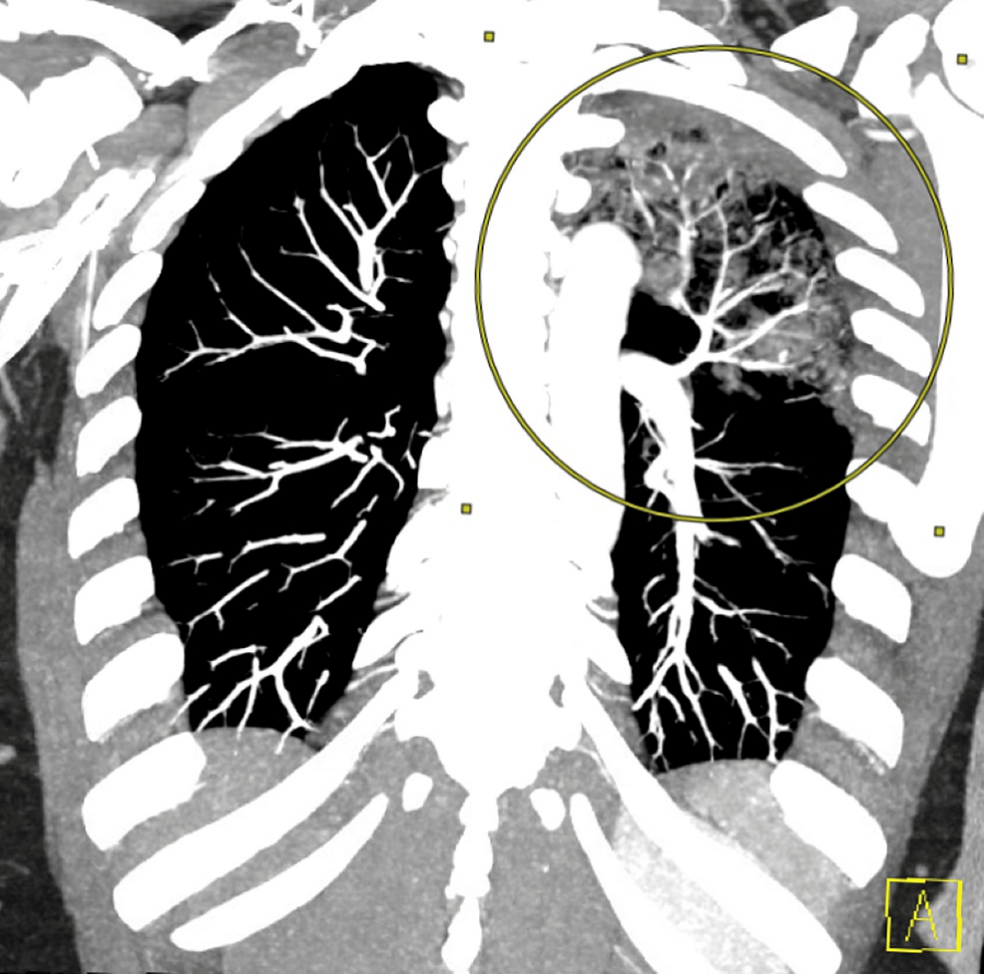
Chest CT with angiography revealing a large left upper lobe pneumonia (yellow circle)

**Figure 2:**
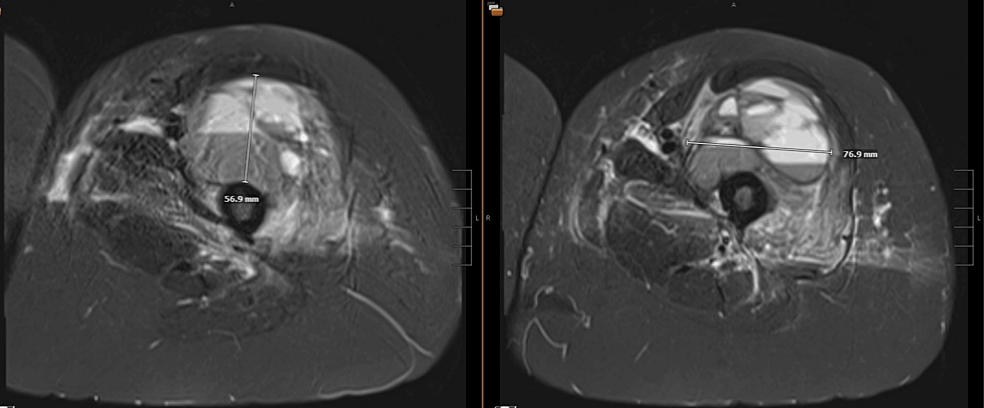
Left femur MRI - Multilobulated multiseptated lesion identified within the anterior compartment musculature centered in the vastus intermedius. The mass measured 76.9 mm x 56.9 mm.

**Day 20**: The patient presented to the emergency department with significant left arm and left leg pain and swelling. She also reported that three days prior to this encounter she had blood-tinged sputum that has since resolved. Lab work revealed an elevated creatine kinase levels at 471 U/L, a high white blood cell count of 12.5 x 10^9^/L, a low red blood cell count of 3.92 million/mm^3^, decreased hemoglobin levels at 11.4 g/dL, a reduced hematocrit of 34%, a mean corpuscular volume of 87 fL, and an elevated platelet count of 511 × 10^9^/L. Computed tomography (CT) angiography revealed a large left upper lobe pneumonia with complex central cavitary density and air-fluid level (Figure 1). These findings were concerning for necrotizing pneumonia. All further infectious disease testing returned normal. An MRI of her left femur was obtained due to the findings of swelling and pain, and it revealed complex lesions in the anterior compartment of thigh and hip adductor musculature (Figure 2). The MRI findings were consistent with complex fluid collection - likely hematoma. No visualized feeding vessels typical of hemangioma and unlikely given acute onset. The patient was admitted to the hospital for cavitary pneumonia and was started on vancomycin and piperacillin/tazobactam. Within the next 3 days, the patient’s hemoglobin decreased from 11.4 to 7.7 g/dL, however, she remained hemodynamically stable. Pulmonology was consulted and suggested that the likely etiology of the pneumonia was chronic aspiration with a low suspicion for atypical infection. The patient was transitioned to amoxicillin/clavulanate potassium upon discharge.

Vascular surgery was consulted for the left thigh hematoma. There was concern that the hematoma was impinging on the femoral nerve due to findings of left lower extremity weakness and anterior thigh numbness. CT angiography of the aorta and bilateral iliofemoral runoffs was obtained and revealed a fluid collection involving the left obturator externus, and left vastus intermedius/lateralis muscles most likely reflecting multifocal hematomas. Masses felt less likely. No evidence of active extravasation. Evacuation of the left thigh hematoma was performed without complications. The operation revealed a large collection of fresh, dark blood that was under pressure. No obvious purulence or soft tissue masses were noted. The patient returned to the OR several days later to undergo wound washout and closure with drain placement. The wound base was clean, and a Jackson-Pratt (JP) drain was placed.

**Day 31**: The patient was deemed stable and medically ready for discharge. The patient was discharged to an inpatient rehab (IPR) facility.

**Figure 3:**
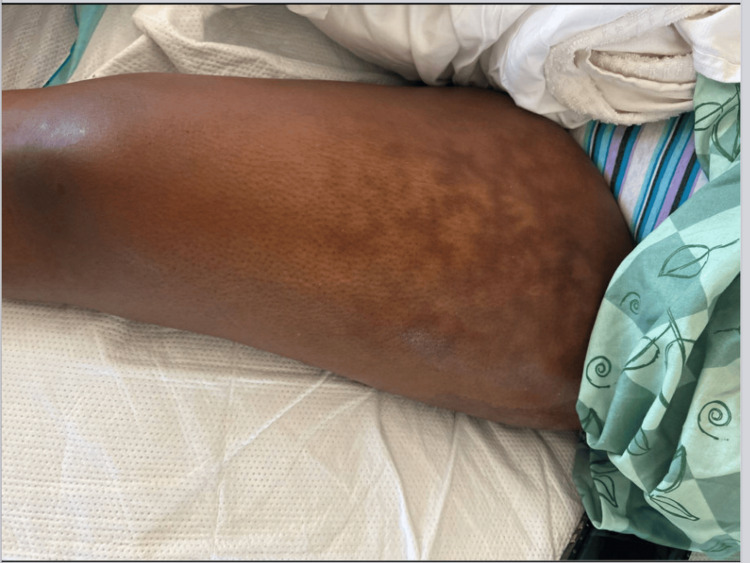
Left thigh - Livedo reticularis

**Day 38**: The patient presented to the emergency department from IPR with a fever, abdominal pain, weakness, and increased output of JP drain in the left thigh. The patient was normotensive and tachycardic. The patient’s lab values revealed a white blood cell count of 19.6 x 10^9^/L, red blood cell count of 1.67 million/mm^3^, hemoglobin of 4.9 g/dL, hematocrit of 15.3%, mean corpuscular volume of 92 fL, platelet count of 443 x 10^9^/L, red cell distribution width (RDW) standard deviation of 50.0 fL, procalcitonin level of 0.18 ng/mL, negative viral PCR respiratory panel, and a positive fecal occult blood test. Physical exam revealed a netlike pattern of red and blue discoloration to the proximal portion of the left thigh, findings consistent with livedo reticularis (Figure 3). A CT angiography with contrast of the chest, abdomen, and pelvis identified several significant findings. Firstly, there is a consolidative airspace opacity at the posterior aspect of the left upper lobe, raising concerns for aspiration or infection. Additionally, a soft tissue mass was observed at the left femur, suggesting a potential evolving hematoma, infection, or malignancy, with a drainage catheter visualized. Furthermore, there is a possible ill-defined mass at the right sartorius muscle, notably larger, and a rim-enhancing area was noted within the left external obturator musculature, larger than in previous imaging. A CT of the left femur with IV contrast revealed multiple rim-enhancing cystic lesions distributed throughout the anterior compartment of the upper left thigh, accompanied by a percutaneous drainage catheter within the substance of the anterior musculature. On MRI, multiple similar-appearing rim-enhancing lesions within the abductor magnus muscle suggest the presence of abscesses. Empiric treatment of sepsis was started, and multiple units of packed red blood cells were given. The patient was admitted to critical care for sepsis likely secondary to abscess of the left thigh. Vascular surgery was consulted.

Vascular surgery determined it was necessary to perform a left thigh wound washout and debridement with vacuum-assisted closure (VAC) placement based on the above findings. The operation was performed without complication. Findings included: left thigh wound with serosanguineous fluid collection - consistent with a seroma, small amount of old blood, and wound dimensions consistent with previous procedure. The wound looked clean with no obvious abscess, infection, or significant active bleeding. The patient was scheduled for a repeat wound washout the following day. Repeat wound washout revealed a deep cavity found extending approximately 10 cm deep in the thigh, down to the femur, which was palpable in the base of the wound. Necrotic tissue was noted in the base of the wound. Some of the necrotic tissue was debrided and sent to pathology. The pathology report on the necrotic tissue revealed a diagnosis of poorly differentiated carcinoma of unknown primary at stage IV, with positive stains for pancytokeratin, cytokeratin 7, and TTF-1.

Oncology was consulted and ordered a CT soft tissue neck with IV Contrast which disclosed a 27.2 mm x 20.4 mm x 34.7 mm mass in the right thyroid. An ultrasound-guided fine-needle aspiration (FNA) of the right thyroid mass resulted in findings consistent with a benign lesion. It was recommended that the patient start radiation therapy to the thigh, consisting of 10 fractions. Although she would qualify for immunotherapy, because she had severe pain and was not stable she was unable to be discharged for outpatient chemotherapy treatment.

Pulmonology was consulted and determined a diagnostic bronchoscopy was indicated due to the patient’s symptoms, findings, and 10-pack-year smoking history. A CT-guided biopsy of the left upper lobe was successfully conducted without complications, and the pathology results indicate a poorly differentiated adenocarcinoma consistent with a primary lung origin. Despite the absence of targetable mutations, the tumor exhibits a high PDL1 expression of 90%. The patient began radiation treatment at this time.

**Figure 4:**
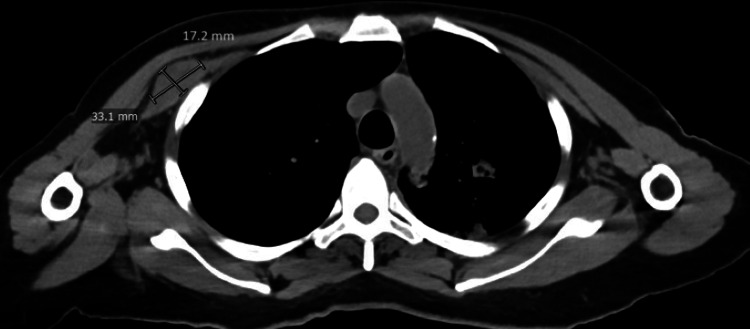
Chest CT - Right pectoralis minor mass measuring 33.1 mm x 17.2 mm

**Day 57**: A repeat CT of the chest showed several concerning findings, including a new mass in the right pectoralis minor (Figure 4), an increased mass in the right supraclavicular region, an enlarged subcutaneous nodule in the left lateral wall, an augmented nodule in the left breast, and heightened mesenteric nodules. There is also concern for lymphangitic spread of the tumor in the left upper lobe and the identification of a new consolidation in the left lower lobe.

**Day 67**: Radiation completed, the patient received 30 Gy in 10 fractions. The patient’s pain was hard to control even with patient-controlled analgesia pump, so a consult to pain management was made.

**Day 76**: The patient had a sudden drop in hemoglobin from 7.5 to 4.9 g/dL with a positive Fecal Occult Blood Test.

**Figure 5:**
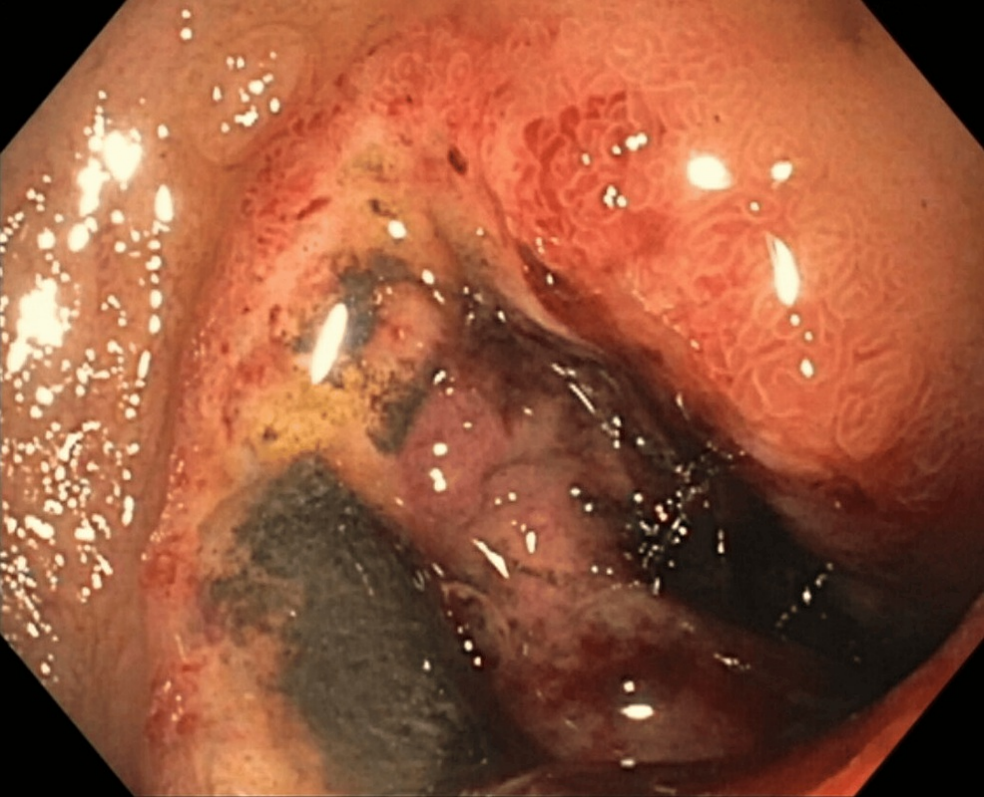
EGD - Fungating and ulcerating mass in duodenal bulb EGD: Esophagogastroduodenoscopy

**Day 81**: The patient reported coffee ground emesis. Esophagogastroduodenoscopy (EGD) performed the following day revealed a fungating and ulcerated mass in the duodenal bulb, covering the whole circumference - causing a gastric outlet obstruction as seen in Figure 5. No biopsy was done at this time. The patient was referred to hospice care.

**Day 88**: The patient suffered a significant GI bleed; the patient expired peacefully with the family at the bedside.

## Discussion

The case presented illustrates an atypical and rapidly progressing presentation of lung adenocarcinoma, a prevalent form of non-small cell lung cancer (NSCLC), that is known for its hematogenous spread to organs like the liver and adrenal glands. The patient initially presented with symptoms suggestive of necrotizing pneumonia and multifocal pain, which later revealed a complex web of underlying conditions including skeletal muscle metastasis (SMM) and a fungating duodenal mass.

Determining the etiology of cavitary lung lesions can be difficult due to overlapping imaging characteristics. It is important to correlate the imaging findings with the clinical history when differentiating between malignant and non-malignant lung lesions. While cavity wall thickness can be indicative (less than 7 mm often suggests benign conditions, whereas greater than 24 mm leans towards malignancy), this is not always the case [4]. The presence of centrilobular nodules tends to indicate a benign disease process while the absence suggests malignancy [4]. Moreover, the degree of contrast enhancement can offer additional diagnostic clues [4]. The combination of symptoms, laboratory results, past medical history, and imaging findings should all be considered when assessing a cavitary lesion with unknown etiology.

When lung adenocarcinoma metastasizes, it generally spreads to the liver and/or the adrenal gland. In this case, the primary site of metastasis was the skeletal muscle, which is rare and often suggests aggressive disease. The mechanical hypothesis, metabolic hypothesis, and immunologic hypothesis are some of several hypotheses that might lend insight as to why skeletal muscle metastasis is rare. The mechanical hypothesis suggests muscle contractions could prevent tumor invasion by creating high pressure and changing blood flow in the tissue [5]. The metabolic hypothesis suggests that the local environment (variable acidity, high lactic acid concentration) of skeletal muscle is unfavorable for the development of tumor foci [6]. The immunologic hypothesis suggests metastatic cells are inhibited by natural killer cells and lymphocytes residing within the skeletal muscle [7].

A retrospective study in 2014 assessed for SMM in 1,754 patients with lung cancer over a five-year period and found it to be prevalent in 46 of the patients. The study found that SMMs were more commonly observed in patients with poorly differentiated and advanced adenocarcinomas. The sites most commonly affected were psoas and buttock muscles [8].

## Conclusions

This case describes an atypical and rapidly progressing presentation of lung adenocarcinoma, a common form of non-small cell lung cancer. While lung adenocarcinoma typically metastasizes to organs such as the liver and adrenal glands, the presented case featured an uncommon metastasis to skeletal muscles which is indicative of aggressive disease. The patient’s condition deteriorated rapidly, and she died within three months of the initial presentation. This case highlights the unpredictable nature of lung adenocarcinoma and the challenges of diagnosing and managing a rapidly progressive disease.
